# The Trp719Arg polymorphism of the KIF6 gene and coronary heart disease risk: systematic review and meta-analysis

**DOI:** 10.1186/s41065-015-0004-7

**Published:** 2015-10-22

**Authors:** David Ruiz-Ramos, Yazmín Hernández-Díaz, Carlos Alfonso Tovilla-Zárate, Isela Juárez-Rojop, María Lilia López-Narváez, Thelma Beatriz González-Castro, Manuel Eduardo Torres-Hernández, Manuel Alfonso Baños-González

**Affiliations:** 1 División Académica de Ciencias de la Salud, Universidad Juárez Autónoma de Tabasco, Villahermosa, Tabasco Mexico; 2 División Académica Multidisciplinaria de Jalpa de Méndez, Universidad Juárez Autónoma de Tabasco, Carretera Cunduacán-Jalpa km. 1, Col. La Esmeralda, C.P. 86690 Cunduacán, Tabasco Mexico; 3 División Académica Multidisciplinaria de Comalcalco, Universidad Juárez Autónoma de Tabasco, Comalcalco, Tabasco Mexico; 4 Hospital General de Yajalón. Secretaría de Salud, Yajalón, Chiapas Mexico; 5 Hospital de Alta Especialidad “Juan Graham Casasús”, Villahermosa, Tabasco Mexico

**Keywords:** Cardiovascular disease, KIF6, Trp719Arg polymorphism, Meta-analysis, Systematic review

## Abstract

**Background:**

Genetic factors play an important role in the pathogenesis of coronary heart disease (CHD). Kinesin-like protein 6 (KIF6) is a new candidate gene for CHD, since it has been identified as a potential risk factor. The aim of this study was to perform a systematic review and meta-analysis of previously published association studies between the Trp719Arg polymorphism of KIF6 and the development of CHD.

**Methods:**

Studies and abstracts investigating the relationship between the Trp719Arg polymorphism of KIF6 and subsequent risk for development of CHD were reviewed. Electronic search from Pubmed and EBSCO databases was performed between 1993 and 2014 to identify studies that fulfilled the inclusion criteria. To analyze the association we used the models: allelic, additive, dominant and recessive. Moreover, we conducted a sub-analysis by populations using the same four models.

**Results:**

Twenty-three studies were included in the meta-analysis. The Trp719Arg polymorphism showed a significant association with CHD when the analysis comprised the population with myocardial infarction (MI) and the additive genetic model was used. Moreover, this polymorphism showed a protective association with CHD when the analysis comprised the whole population using the recessive genetic model.

**Conclusions:**

Our findings indicate that the Trp719Arg polymorphism of the KIF6 gene is an important risk factor for developing MI and that allele 719Arg may have a protective association to present CHD in all populations.

**PROSPERO registration:**

CRD42015024602.

**Electronic supplementary material:**

The online version of this article (doi:10.1186/s41065-015-0004-7) contains supplementary material, which is available to authorized users.

## Background

The risk for coronary heart disease (CHD) is influenced by both environmental and genetic factors and often it is initially detected from clinical manifestations such as angina, myocardial infarction or sudden death due to artery occlusion [1]. Several environmental factors, such as obesity, oxidative stress, alcoholism, smoking and lack of exercise have been identified as risk factors for these diseases. In recent years, multiple genetic analysis studies have identified several loci and variants that are strongly associated with CHD [2, 3]. Kinesin-like protein 6 (KIF6) is considered a candidate gene for CHD, since it has been identified as a potential risk factor in European populations [4, 5]. KIF6 is a member of a family of molecular motors involved in intracellular transport of protein complexes, membrane organelles, and messenger ribonucleic acid along microtubules. This gene spans a genomic region of about 390,000 base pairs at human chromosome 6p21; moreover, it is ubiquitously expressed in coronary arteries and other vascular tissue [6, 7]. To date, multiple large prospective and case–control studies have reported an association of a common KIF6 gene polymorphism—Trp719Arg single nucleotide polymorphism (SNP) (rs20455)— with CHD risk. Carriers of the 719Arg allele exhibit a 50 % increased risk of events compared with non-carriers [5, 8]. However, some studies have not verified this conclusion. In view of the discrepancies in the findings of previous published studies, we aimed to perform a systematic review and meta-analysis to clarify the association between Trp719Arg in KIF6 and CHD to get a better understanding of this relationship.

## Methods

The meta-analysis and systematic review were performed by following the Preferred Reporting Items for Systematic Reviews and Meta-Analyses (PRISMA) criteria [9, 10]. The PRISMA checklist is included as Additional file 1. PROSPERO registration: CRD42015024602.

### Identification and selection of publications

To perform the meta-analysis, we systematically searched for available articles in multiple electronic databases. The literature search was conducted using PubMed and EBSCO databases. Relevant studies were identified using the terms: “Kinesin 6 AND polymorphisms AND cardiovascular heart disease”, “KIF6 AND polymorphisms AND cardiovascular heart disease”, “KIF6 AND polymorphisms AND CHD”, “KIF6 AND Trp719Arg AND cardiovascular heart disease” and “KIF6 AND Trp719Arg AND CHD”. These words were combined to retrieve the summaries. The search also implicated the review of the bibliography cited at the end of the various research articles.

### Inclusion and exclusion criteria

Two researchers (González-Castro and Hernández-Díaz) working independently screened each of the titles, abstracts and full texts to determine inclusion. When the researchers were in disagreement a third researcher (Tovilla-Zárate) was consulted. Studies were included if they met the following criteria: (1) to be published in peer-reviewed journals, (2) to have a case–control study design, (3) to contain independent data, (4) to be association studies in which the frequencies of three genotypes were clearly stated or could be calculated, (5) to include diagnosis of a cardiovascular disease in the patient study group, and (6) the articles had to be written in English. Studies were excluded when: (1) they were not case–control studies, (2) they were reviews, comments or editorial articles, (3) provided insufficient data, and (4) they were repeated studies.

### Data extraction

The same authors mentioned previously extracted the information from all the included reports and reached consensus on all the items. The following data were obtained from each of the studies: authors, year of publication, location, ethnic group, number of cases and/or controls, age, gender and cardiovascular diagnosis of the participants. If these data were not available in the studies, the corresponding author of the respective article was contacted.

### Evaluation of statistical associations

For the meta-analysis, the odds ratio (OR) and 95 % confidence interval (CI) values were estimated and used to evaluate the strength of the association of KIF6 Trp719Arg polymorphisms with CHD risk. Pooled ORs were calculated following four genetic models: dominant (A/G + A/A vs G/G), recessive (G/G + A/G vs A/A), additive (A/A vs G/G) and allelic (A vs G). The EPIDAT 3.1 program (http://dxsp.sergas.es) used for this part is freely available for epidemiologic analysis of tabulated data. On the other hand, to explore the problem of publication bias, the Egger’s test and funnel plots were calculated with the same software. This last approach standardizes the effect of each of the published studies on the vertical axis and its correspondent precision on the horizontal axis. Sample heterogeneity was analyzed with the Dersimonian and Laird’s Q test. Q test results were complemented with graphs to help the visualization of those studies favoring heterogeneity. For all these procedures we used the above-mentioned program. Moreover, to aboard the problem of a small sample size we performed a meta-regression based in ages; this analysis was carried out in the comprehensive meta-analysis software version 2. Next, a chi-squared (*χ* ^2^) analysis was used to calculate the Hardy-Weinberg equilibrium to evaluate genotype distribution. In order to strengthen the analysis we evaluated publication bias by using the GRADE approach and assessed the risk of bias. The Newcastle-Ottawa Assessment Scale (NOS) was used for inclusion in the systematic review by scoring the methodological quality. We established a score of six as cut-off point to distinguish high from low quality studies [11] (see Additional file 2).

## Results

### Studies included in the meta-analysis

**Fig. 1:**
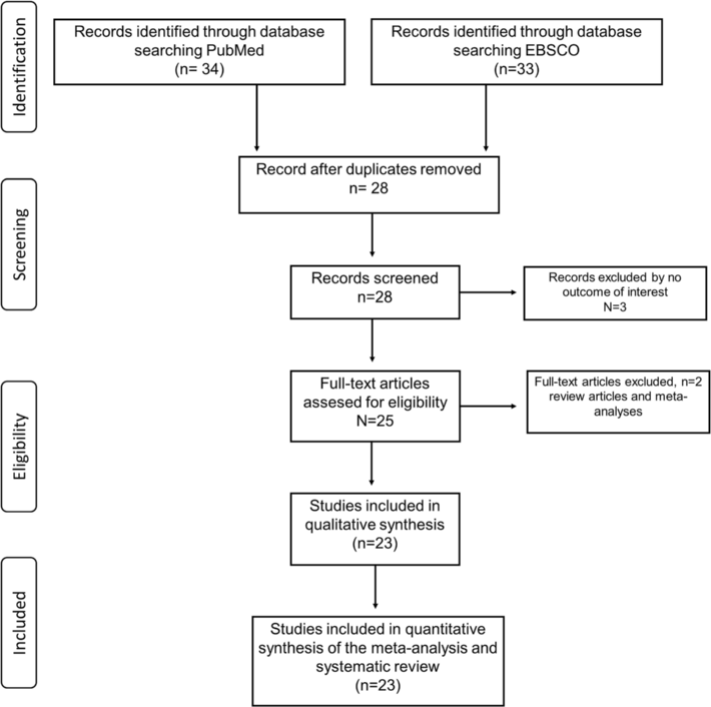
Flow-chart showing the search strategy and inclusion/exclusion criteria used in the meta-analysis and systematic review

The electronic searches yielded 34 potentially relevant studies. From these 6 reports were excluded because they consisted of duplicated publications, hence 28 studies were potentially relevant for inclusion in our study; 5 studies were further excluded because either the Trp719Arg genotype was not detected or the studies did not present a control population. In the end, a total of 23 articles met the inclusion criteria (Fig. 1) [12, 13]. The overall study population included in the current meta-analysis consisted of 38,906 subjects, of which 17,812 were cases and 21,094 controls. We divided the included studies into sub-groups according to their diagnosis: coronary artery diseases (CAD) population (5235 patients, 6682 controls) and myocardial infarction (MI) population (12,577 patients, 14,412 controls), and another section in accordance with ethnicity: Caucasians (12,897 patients, 14,897 controls). The characteristics of the included studies are summarized in Tables 1 and 2. The included studies (*n* = 23) were published between 1993 and 2014.

**Table 1:** Descriptive characteristics of the association studies on the Trp719Arg polymorphism of the KIF6 gene and CHD included in the meta-analysis and systematic review

Author	Year	Country	Ethnicity	Number		Diagnosis	Mean age	
				Cases	Control		Cases	Control
Berglund, G. [17]	1993	Sweden	Caucasians	86	99	MI	48.5	48.7
Vartiainen, E. [18]	2000	Finland	Caucasians	167	172	MI	47.1	47.1
Senti, M. [19]	2001	Spain	Caucasians	312	317	MI	45.9	46.0
Yusuf, S. [20]	2004	Bangladesh, Sri Lanka, Pakistan, others.	Asians	1092	1187	MI	51.4	49.8
Low, A. F. [21]	2005	USA	Caucasians	204	260	MI	47.0	53.8
Helgadottir, A. [22]	2007	USA	Caucasians	875	447	CAD	48.9	59.8
Helgadottir, A. [22]	2007	USA	Caucasians	933	468	CAD	52.7	61.7
Samani, N. J. [23]	2007	Germany	Caucasians	1126	1277	MI	51.3	51.2
Samani, N. J. [23]	2007	Germany	Caucasians	722	1643	MI	50.2	62.5
Meng, W. [24]	2007	Northern Ireland	Caucasians	482	622	MI	46.0	55.2
Iakoubova, O. [14]	2008	USA	Caucasians	276	519	MI	56.4	56.2
Meiner, V. [25]	2008	USA	Caucasians	505	559	MI	46.0	45.2
Serre, D. [26]	2008	Several	Several	789	859	MI	61.6	61.2
Morgan, T. M. [27]	2008	USA	Caucasians	807	637	MI	61.5	60.7
Assimes, T. L. [28]	2008	USA	Caucasians	505	514	CAD	45.4	45.6
Vennemann, M. M. [29]	2008	Germany	Caucasians	793	1121	MI	52.2	52.6
Sutton, B. S. [30]	2008	USA	Caucasians	1575	970	MI	28.9	52.4
Martinelli, W. [31]	2008	Italy	Caucasians	1106	383	CAD	61.4	58.0
Herrera-Galeano, J. E. [32]	2008	USA	Caucasians	378	2652	CAD	46.9	47.2
Stewart, A. F. [33]	2009	Canada	Caucasians	1540	1455	MI	49.0	75
Luke, M. M. [34]	2009	Austria	Caucasians	505	782	CAD	66.0	58.8
Bare, L. A. [13]	2010	Costa Rica	Latin-Americans	1987	2147	MI	58.3	58.3
Wu, G. [16]	2012	China	Asians	356	568	CAD	64.9	60.3
Wu, G. [16]	2012	China	Asians	114	568	MI	64.9	60.3
Peng, P. [15]	2012	China	Asians	289	522	CAD	-	-
Wu, G. [35]	2014	China	Asians	288	346	CAD	63.8	60.2

**Table 2:** Genotype and allele distribution in association studies on the Trp719Arg polymorphism of the KIF6 gene with CHD

Author	Genotype cases			Genotype controls			Allele cases		Allele controls		HWE	
	Trp/Trp	Trp/Arg	Arg/Arg	Trp/Trp	Trp/Arg	Arg/Arg	Trp	Arg	Trp	Arg	Cases *p* value	Controls *p* value
Berglund, G. [17]	35	38	13	33	54	12	108	64	120	78	0.64	0.20
Vartiainen, E. [18]	64	81	22	73	76	23	209	125	222	122	0.74	0.62
Senti, M. [19]	134	139	39	141	137	39	407	217	419	215	0.80	0.53
Yusuf, S. [20]	351	498	243	389	531	267	1200	984	1309	1065	0.09	0.06
Low, A. F. [21]	89	86	29	114	111	35	264	144	339	181	0.53	0.34
Helgadottir, A. [22]	370	399	106	174	221	52	1139	611	569	325	0.94	0.18
Helgadottir, A. [22]	359	441	133	194	213	61	1159	707	601	335	0.59	0.84
Samani, N. J. [23]	447	529	150	522	593	162	1423	829	1637	917	0.79	0.80
Samani, N. J. [23]	293	328	101	662	753	228	914	530	2077	1209	0.57	0.55
Meng, W. [24]	203	226	53	261	292	69	632	332	814	430	0.42	0.37
Iakoubova, O. [14]	104	137	35	256	204	59	345	207	716	322	0.36	0.06
Meiner, V. [25]	187	228	90	216	260	83	602	408	692	426	0.16	0.78
Serre, D. [26]	335	337	117	354	402	103	1007	571	1110	608	0.03*	0.55
Morgan, T. M. [27]	322	377	108	256	304	77	1021	593	816	458	0.93	0.39
Assimes, T. L. [28]	162	187	83	144	183	130	511	353	471	443	0.03*	0.00*
Vennemann, M. M. [29]	311	379	103	430	528	163	1001	585	1388	854	0.44	1.00
Sutton, B. S. [30]	545	570	183	297	347	86	1660	936	941	519	0.09	0.33
Martinelli, W. [31]	437	501	168	145	191	47	1375	837	481	285	0.22	0.22
Herrera-Galeano, J. E. [32]	106	148	31	626	752	201	360	210	2004	1154	0.05	0.30
Stewart, A. F. [33]	183	695	662	205	616	634	1061	2019	1026	1884	1.00	0.00*
Luke, M. M. [34]	73	254	178	102	373	307	400	610	577	987	0.26	0.53
Bare, L. A. [13]	785	952	250	896	966	285	2522	1452	2758	1536	0.13	0.33
Wu, G. [16]	104	164	88	168	268	132	372	340	604	532	0.16	0.20
Wu, G. [16]	16	68	30	168	268	132	100	128	604	532	0.03*	0.20
Peng, P. [14]	69	149	71	139	262	121	287	291	540	504	0.63	0.93
Wu, G. [35]	74	141	73	101	166	79	289	287	368	324	0.72	0.51

*p value: statistical significance

### Analysis of the association between the KIF6 Trp719Arg polymorphism and CHD in all populations

**Fig. 2:**
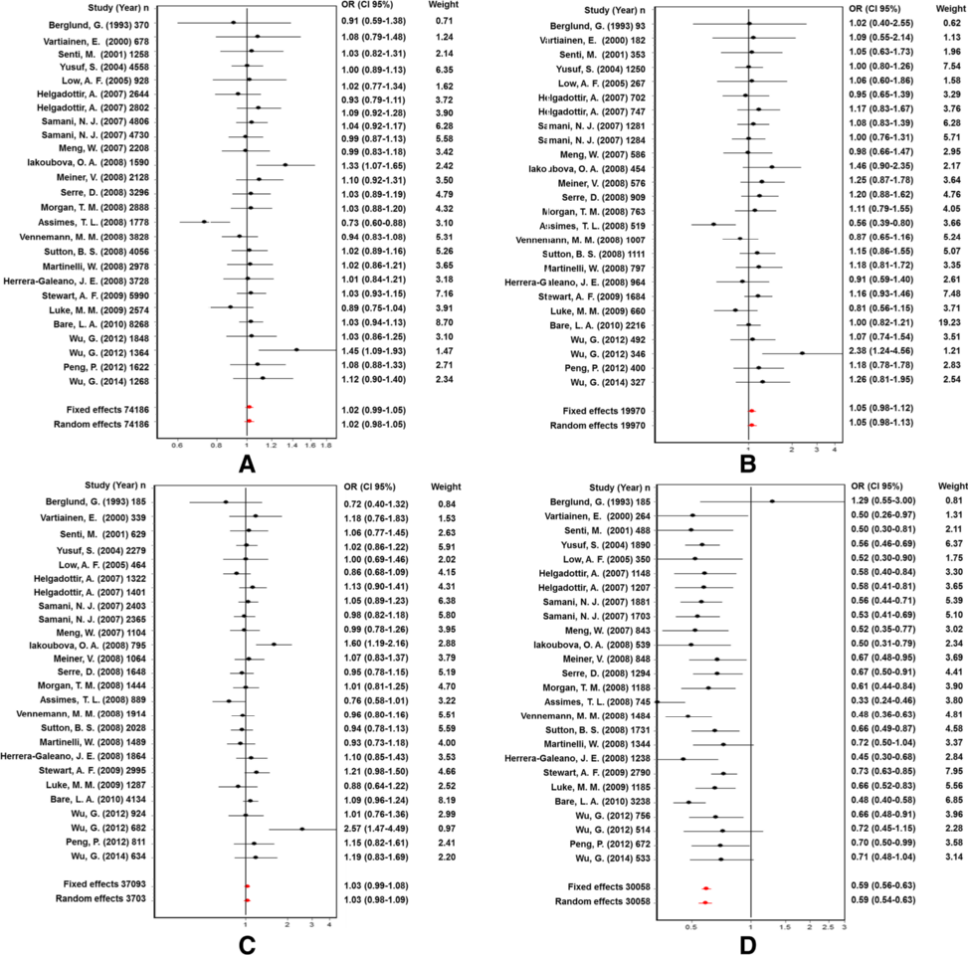
Odds ratios and forest plots of the Trp719Arg polymorphism in overall studies without heterogeneity using the following models: **a**) Allelic, **b**) Additive, **c**) Dominant and **d**) Recessive

**Fig. 3:**
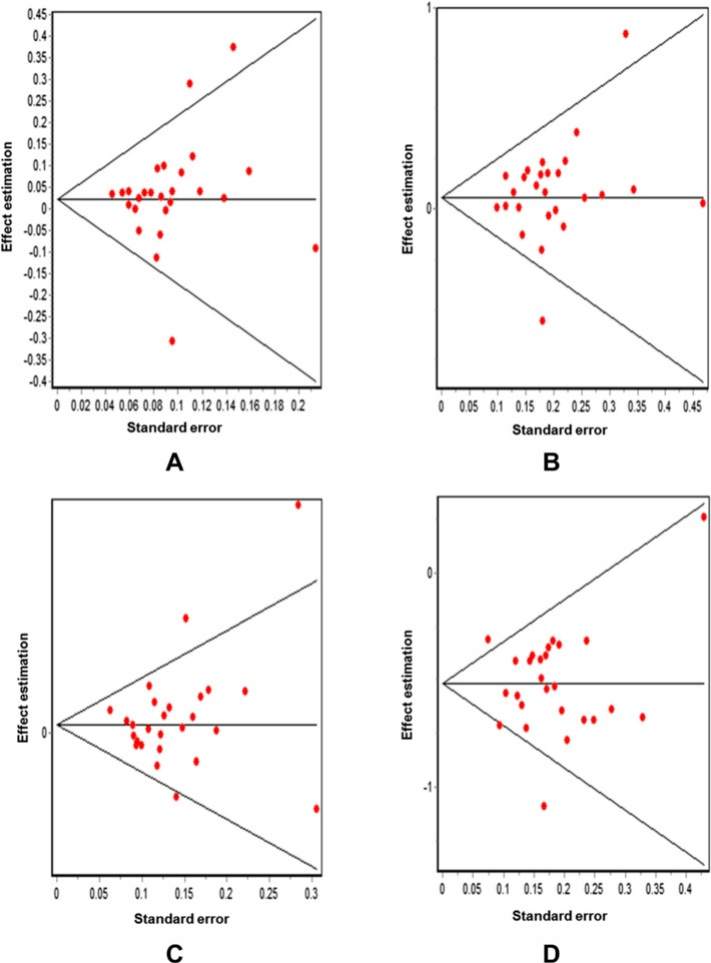
Egger’s funnel plots in overall studies indicating publication bias in studies on CHD and the Trp719Arg polymorphism without heterogeneity using the following models: **a**) Allelic; **b**) Additive; **c**) Dominant, and **d**) Recessive

**Fig. 4:**
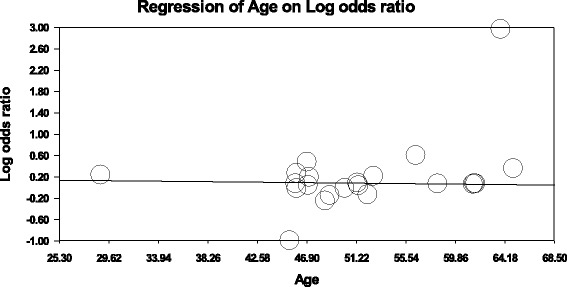
Meta-regression plot showing relationship between age and the log odds ratio in all populations

The association between the Trp719Arg polymorphism and susceptibility to CHD was analyzed in 23 independent studies. The results of the meta-analysis in the correlation between CHD and Trp719Arg polymorphism in 23 case–control studies are shown in Fig. 2. There was no inter-study heterogeneity among overall studies of the Trp719Arg polymorphism in all four genetic models (allelic, additive, dominant and recessive). We used the random-effects model, which yielded a slight association in the recessive genetic model (OR: 0.59, 95 % CI: 0.54–0.63; Q test: 0.05; Egger’s test: 0.71). However, no significant association was observed when all the populations were considered in the analysis using the other genetic models (allelic: OR: 1.02, 95 % CI: 0.98–1.05; Q test: 0.16; Egger’s test: 0.45; additive: OR: 1.05, 95 % CI: 0.98–1.13; Q test: 0.24; Egger’s test: 0.33, and dominant: OR: 1.03, 95 % CI: 0.98–1.09; Q test: 0.06; Egger’s test: 0.39) (Tables 3 and 4; Fig. 3). With regard to the meta-regression performed based on the ages of the whole population, the analysis revealed a point estimate slope of 0.00212 and a *p*-value of 0.722 (Fig. 4).

**Table 3:** Analysis of association studies on the Trp719Arg polymorphism of the KIF6 gene with CHD in all populations, CAD populations, MI populations and Caucasian populations in this study

Model analysis		All populations			CAD			MI		
		Random effects OR (CI 95 %)	*P* value of Q test	*P* value of Egger’s test	Random effects OR (CI 95 %)	*P* value of Q test	*P* value of Egger’s test	Random effects OR (CI 95 %)	*P* value of Q test	*P* value of Egger’s test
Allelic	With heterogeneity	-	-	-	-	-	-			
	Without heterogeneity	1.02(0.98–1.05)	0.16	0.45	0.98(0.90–1.07)	0.05	0.58	1.03(0.99–1.07)	0.59	0.14
Additive	With heterogeneity	-	-	-	-	-	-			
	Without heterogeneity	1.05(0.98–1.13)	0.24	0.33	0.98(0.82–1.16)	0.06	0.32	1.08(1–00–1.16)	0.69	0.10
Dominant	With heterogeneity	-	-	-	-	-	-			
	Without heterogeneity	1.03(0.98–1.09)	0.06	0.39	0.98(0.89–1.08)	0.32	0.63	1.06(0.98–1.13)	0.05	0.23
Recessive	With heterogeneity	-	-	-	-	-	-			
	Without heterogeneity	0.59(0.54–0.63)	0.05	0.71	0.97(0.83–1.13)	0.05	0.35	1.03(0.96–1.10)	0.89	0.06

**Table 4:** Analysis of association studies on the Trp719Arg polymorphism of the KIF6 gene with CHD in all populations, CAD populations, MI populations and Caucasian populations in this study

Model analysis		Caucasians		
		Random effects OR (CI 95 %)	*P* value of Q test	*P* value of Egger’s test
Allelic	With heterogeneity	-	-	-
	Without heterogeneity	1.00(0.96–1.05)	0.14	0.99
Additive	With heterogeneity	-	-	-
	Without heterogeneity	1.02(0.94–1.12)	0.28	0.87
Dominant	With heterogeneity	-	-	-
	Without heterogeneity	1.01(0.95–1.08)	0.18	0.99
Recessive	With heterogeneity	-	-	-
	Without heterogeneity	1.00(0.92–1.08)	0.23	0.48

### Analysis of the association between the KIF6 Trp719Arg polymorphism and CHD in CAD populations

**Fig. 5:**
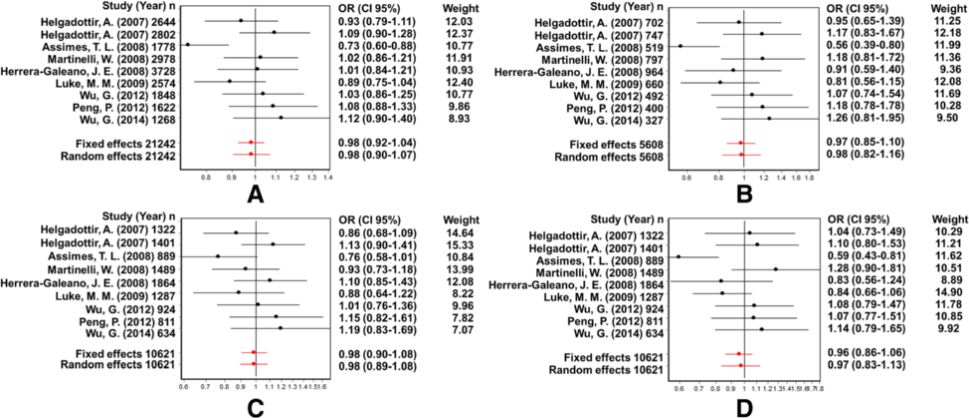
Odds ratios and forest plots of the Trp719Arg polymorphism in CAD population without heterogeneity using the following models: **a**) Allelic, **b**) Adittive, **c**) Dominant and **d**) Recessive

**Fig. 6:**
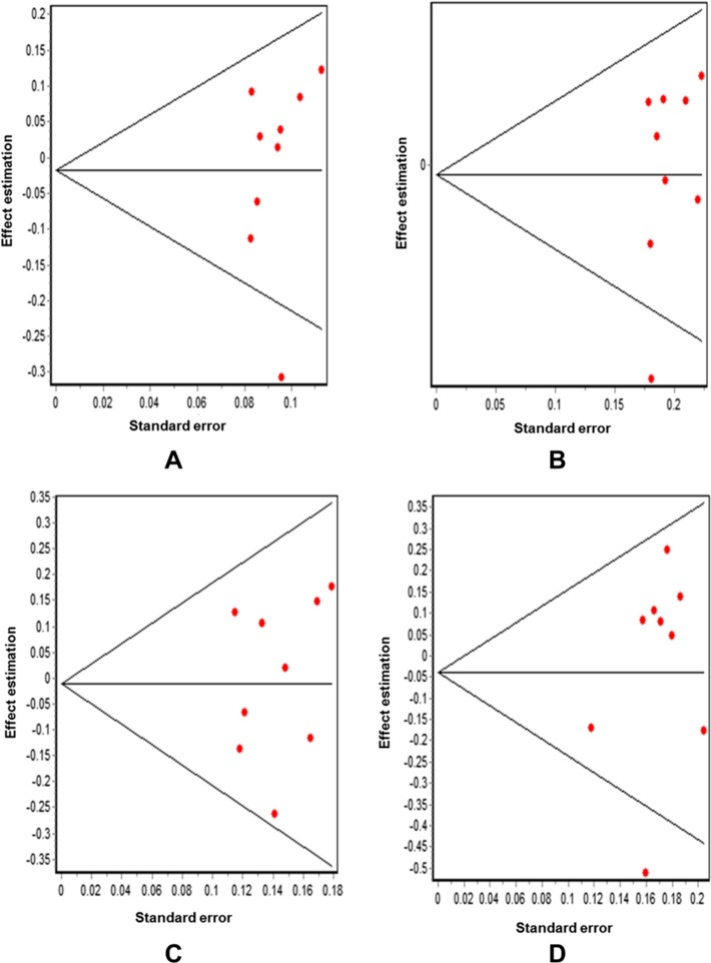
Egger’s funnel plots of the Trp719Arg polymorphism indicating publication bias in CAD population without heterogeneity using **a**) Allelic, **b**) Adittive, **c**) Dominant and **d**) Recessive

Information about 9 cases of coronary artery diseases (CADs) was available in 5235 patients. The analysis of CAD populations did not show a significant association between the Trp719Arg polymorphism of the KIF6 gene and CHD using the random effects model in all four genetic models (allelic model: OR: 0.98, 95 % CI: 0.90–1.07; Q test: 0.05; Egger’s test: 0.58; additive model: OR: 0.98, 95 % CI: 0.82–1.16; Q test: 0.06; Egger’s test: 0.32; dominant model: OR: 0.98, 95 % CI: 0.89–1.08; Q test: 0.32; Egger’s test: 0.63, and recessive model: OR: 0.97, 95 % CI: 0.83–1.13; Q test: 0.05; Egger’s test: 0.35) (Table 3; Figs. 5 and 6).

### Analysis of the association between the KIF6 Trp719Arg polymorphism and CHD in MI populations

**Fig. 7:**
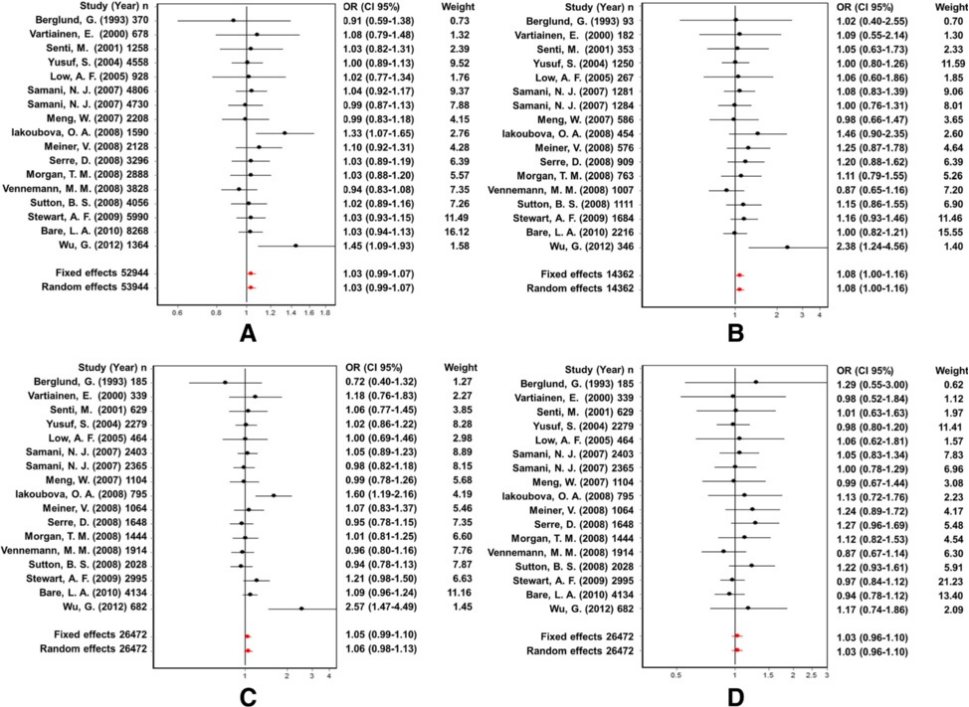
Odds ratios and forest plots of the Trp719Arg polymorphism in MI population without heterogeneity using the following models: **a**) Allelic, **b**) Adittive, **c**) Dominant and **d**) Recessive

**Fig. 8:**
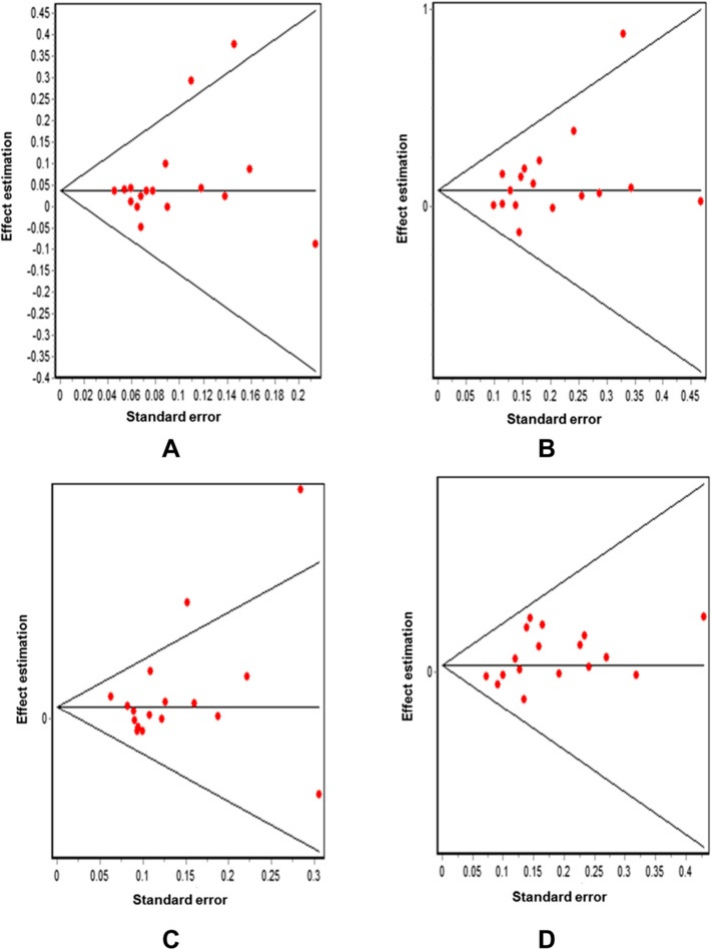
Egger’s funnel plots of the Trp719Arg polymorphism indicating publication bias in MI population without heterogeneity using **a**) Allelic, **b**) Adittive, **c**) Dominant and **d**) Recessive

**Fig. 9:**
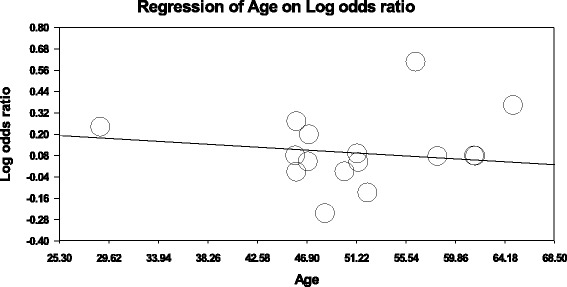
Meta-regression plot showing relationship between age and the log odds ratio in MI population

Subsequently, we explored this SNP in patients with MI and the analysis indicated that the Trp719Arg polymorphism of the KIF6 gene was significantly associated with CHD only in the additive model (Random effects: OR: 1.08, 95 % CI: 1.00–1.16; Q test: 0.69; Egger’s test: 0.10) (Table 3; Figs. 7 and 8). The other genetic models did not show a significant association between these two parameters (allelic: OR: 1.03, 95 % CI: 0.99–1.07; Q test: 0.59; Egger’s test: 0.14; dominant: OR: 1.06, 95 % CI: 0.98–1.13; Q test: 0.05; Egger’s test: 0.23, and recessive: OR: 1.03, 95 % CI: 0.96–1.10; Q test: 0.89; Egger’s test: 0.06) (Table 3; Figs. 7 and 8). On the other hand, the meta-regression analysis based on the age of the patients who had MI showed a point estimate slope of 0.00379 and a *p*-value of 1.242 (Fig. 9).

### Analysis of the association between the KIF6 Trp719Arg polymorphism and CHD in Caucasian populations

**Fig. 10:**
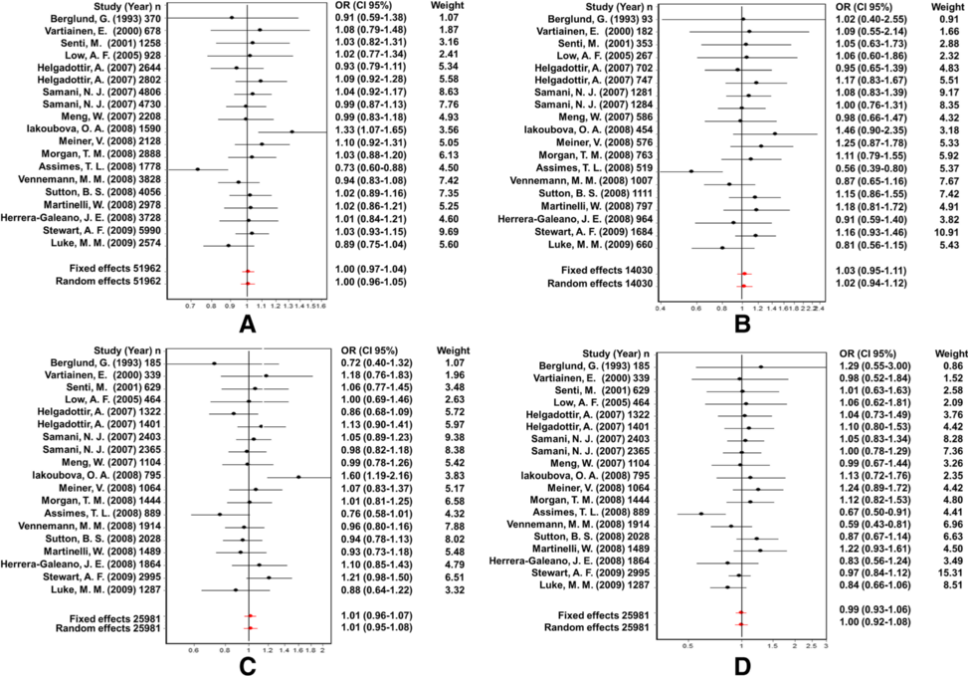
Odds ratios and forest plots of of the Trp719Arg polymorphism in Caucasian population without heterogeneity using the following models: **a**) Allelic, **b**) Additive, **c**) Dominant and **d**) Recessive

**Fig. 11:**
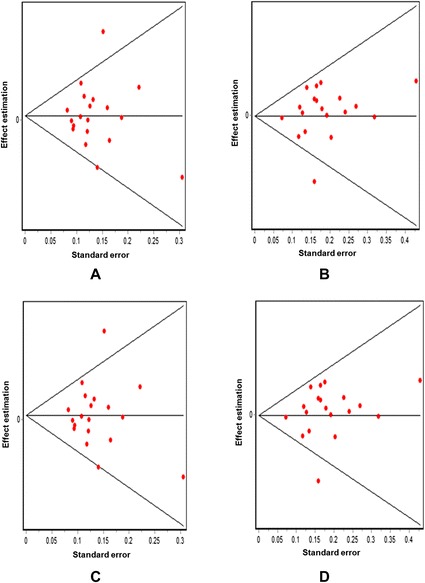
Egger’s funnel plots of the Trp719Arg polymorphism indicating publication bias in Caucasian population without heterogeneity using the following models: **a**) Allelic, **b**) Additive, **c**) Dominant and **d**) Recessive

The analysis in Caucasian populations did not show a significant association between the Trp719Arg polymorphism of the KIF6 gene and CHD in all four genetic models (allelic model: OR: 1.00, 95 % CI: 0.96–1.05; Q test: 0.14; Egger’s test: 0.99; additive model: OR: 1.02, 95 % CI: 0.94–1.12; Q test: 0.28; Egger’s test: 0.87; dominant model: OR: 1.01, 95 % CI: 0.95–1.08; Q test: 0.18; Egger’s test: 0.99, and recessive model: OR: 1.00, 95 % CI: 0.92–1.08; Q test: 0.23; Egger’s test: 0.48) (Table 4; Figs. 10 and 11).

## Discussion

In this study, we explored the relationship between the Trp719Arg polymorphism of the KIF6 gene and CHD given that several lines of evidence have shown an association between the Trp719Arg polymorphism and increased risk of CHD in the placebo groups of some clinical trials such as the Cholesterol and Recurrent Events (CARE) study and the West of Scotland Coronary Prevention Study (WOSCOPS) [14]. Moreover this polymorphism has been also associated with risk to developing various CHDs in prospective population-based reports such as the Atherosclerosis Risk in Communities (ARIC) study [1], Cardiovascular Health Study (CHS) [12] and the Women’s Health Study (WHS) [7] that encompass a broad spectrum of populations. With this evidence, we performed a meta-analysis and systematic review to evaluate genetic associations between the Trp719Arg polymorphism of the KIF6 gene and susceptibility to manifesting some CHD. 23 studies with a total of 38,906 subjects were eligible. We conducted four principal analyses in the present work. The first one involved whole populations, the second comprised CAD individuals, the third MI individuals and the last ethnicity (Caucasians). It is worth noting that none of the carried out analyses showed heterogeneity, therefore, this meta-analysis was conducted with a group of studies that was homogeneous in clinical and methodological terms and provided a meaningful sample. In the overall analysis, we found that in all the studies concerning this variant; the pooled ORs suggested a possible protective role to present CHD clinically. In the stratified analysis by CAD, we could not find any significant association in all the analyses performed. However, we found a possible relation between the Trp719Arg polymorphism of the KIF6 gene and CHD in recessive genetic models of the MI subgroup. Additionally, we conducted a meta-regression analyses, where the age role in the heterogeneity among studies in MI sample as well as the whole sample population was explored; this analyses showed the same outcomes obtained in meta-analysis. Finally, we did other analysis considering only Caucasian populations and we could not find any significant association in all the analyses. Similarly, the meta-analysis failed to find a significant relationship between Trp719Arg and the risk of CHD in Caucasian populations [15]. There are several explanations for the present outcomes concerning the lack of association of Trp719Arg with CHD. First, there are differences in diagnosis in the population of patients and, second, the discrepancy of association between populations may be attributed to different genetic backgrounds and environmental factors [13, 16].

Our findings demonstrate that this polymorphism may be a risk for heart disease development. In addition, some limitations of the present meta-analysis must be addressed. First, we performed the present meta-analysis based only on published studies. We consider that by selecting only published results, we ensure that our meta-analysis excludes poorly designed studies. Second, although the present analysis involves 23 studies, it is relatively small in comparison with other meta-analyses on different diseases. However this limitation involved a meta-regression analysis in order to resolve the problem. We consider that the number of eligible studies included in our meta-analysis is small, hence to validate our results a larger number of studies must be included in future investigations. In the sub-group analysis by ethnicity, we only included Caucasian populations but we acknowledge the importance of theTrp719Arg polymorphism for CHD development in Asian populations. We suggest that more studies investigating this association must be undertaken in Asian populations.

## Conclusions

The present study has demonstrated that the Trp719Arg polymorphism of the KIF6 gene is an important risk factor for developing MI. Moreover, our findings suggest that allele 719Arg may exert a protective association to present CHD in all populations.

## Additional files

Additional file 1:
**Prisma 2009 Checklist.** (PDF 214 kb)
Additional file 2: Table S1. Methodological quality of KIF6 gene studies association included based on the Newcastle-Ottawa scale. **Table S2.** Summary finding of studies association between KIF6 gene and CHD. (PDF 270 kb)
