# Supplementary material for: The Trp719Arg polymorphism of the KIF6 gene and coronary heart disease risk: systematic review and meta-analysis
Source: Hereditas. 2015 Oct 22;152:3. doi: 10.1186/s41065-015-0004-7 (PMC5224589; doi:10.1186/s41065-015-0004-7)
Supplement: Additional file 2: Table S1. — Methodological quality of KIF6 gene studies association included based on the Newcastle-Ottawa scale. Table S2. Summary finding of studies association between KIF6 gene and CHD. (PDF 270 kb) [file 41065_2015_4_MOESM2_ESM.pdf]

Table 1. Methodological quality of KIF6 gene studies association included based on the Newcastle-Ottawa scale

Reference	Selection	Comparability	Exposure	Total score
Berglund, G.[17]	☆☆	☆☆	☆ ☆	6
Vartiainen, E. [18]	☆☆☆	☆☆	☆☆☆	8
Senti, M.[19]	☆☆	☆	☆☆☆	6
Yusuf, S.[20]	☆☆☆	☆	☆☆☆	7
Low, A. F.[21]	☆☆	☆☆	☆☆	6
Helgadottir, A.[22]	☆☆☆	☆☆	☆☆	7
Samani, N. J.[23]	☆☆☆	☆	☆☆	6
Meng, W.[24]	☆☆☆	☆	☆☆☆	7
Iakoubova, O.[12]	☆☆	☆☆	☆☆	6
Meiner, V.[25]	☆☆☆	☆	☆☆☆	7
Serre, D.[26]	☆☆	☆	☆☆☆	6
Morgan, T. M.[27]	☆☆☆	☆☆	☆☆☆	8
Assimes, T. L.[28]	☆☆☆	☆☆	☆☆	7
Vennemann, M. M.[29]	☆☆☆	☆	☆☆☆	7
Sutton, B. S.[30]	☆☆	☆☆	☆☆	6
Martinelli, W.[31]	☆☆	☆☆	☆☆	6
Herrera-Galeano, J. E.[32]	☆☆	☆☆	☆ ☆	6
Stewart, A. F.[33]	☆☆☆	☆	☆☆☆	7

Luke, M. M.[34]	☆☆	☆	☆☆☆	6
Bare, L. A.[15]	☆☆☆	☆☆	☆☆☆	8
Wu, G.[16]	☆☆	☆☆	☆☆	6
Peng, P.[14]	☆☆☆	☆☆	☆☆	7
Wu, G.[35]	☆☆☆	☆	☆☆☆	7

Table 2. Summary finding of studies association between KIF6 gene and CHD.

Reference	OR (CI 95%)	Number of patients (studies)	Design	Quality evidence (GRADE)	Publication bias
Berglund, G.[17]	0.91 (0.59-1.38)	86	Case-control	⨁⨁⨁○ Moderate	Undetected
Vartiainen, E. [18]	1.08 (0.79-1.48)	167	Case-control	⨁⨁⨁○ Moderate	Undetected
Senti, M.[19]	1.03 (0.82-1.31)	312	Case-control	⨁⨁⨁○ Moderate	Undetected
Yusuf, S.[20]	1.00 (0.89-1.13)	1092	Case-control	⨁⨁⨁○ Moderate	Undetected
Low, A. F.[21]	1.02 (0.77-1.34)	204	Case-control	⨁⨁⨁⨁ High	Undetected
Helgadottir, A.[22]	0.93 (0.79-1.11)	875	Case-control	⨁⨁⨁○ Moderate	Undetected
Samani, N. J.[23]	1.04 (0.92-1.17)	1126	Case-control	⨁⨁⨁○ Moderate	Undetected
Meng, W.[24]	0.99 (0.83-1.18)	482	Case-control	⨁⨁⨁○ Moderate	Undetected
Iakoubova, O.[12]	1.33 (1.07-1.65)	276	Case-control	⨁⨁⨁○ Moderate	Undetected
Meiner, V.[25]	1.10 (0.92-1.31)	505	Case-control	⨁⨁⨁○ Moderate	Undetected
Serre, D.[26]	1.03 (0.89-1.19)	789	Case-control	⨁⨁⨁○ Moderate	Undetected
Morgan, T. M.[27]	1.03 (0.88-1.20)	807	Case-control	⨁⨁⨁○ Moderate	Undetected
Assimes, T. L.[28]	0.73 (0.60-0.88)	505	Case-control	⨁⨁⨁○ Moderate	Undetected
Vennemann, M. M.[29]	0.94 (0.83-1.08)	793	Case-control	⨁⨁⨁⨁ High	Undetected
Sutton, B. S.[30]	1.02 (0.89-1.16)	1575	Case-control	⨁⨁⨁⨁ High	Undetected
Martinelli, W.[31]	1.02 (0.86-1.21)	1106	Case-control	⨁⨁⨁○ Moderate	Undetected
Herrera-Galeano, J. E.[32]	1.01 (0.84-1.21)	378	Case-control	⨁⨁⨁○ Moderate	Undetected

Stewart, A. F.[33]	1.03 (0.93-1.15)	1540	Case-control	⊕⊕⊕⊕ High	Undetected
Luke, M. M.[34]	0.89 (0.75-1.04)	505	Case-control	⊕⊕⊕○ Moderate	Undetected
Bare, L. A.[15]	1.03 (0.94-1.13)	1987	Case-control	⊕⊕⊕○ Moderate	Undetected
Wu, G.[16]	1.03 (0.86-1.25)	356	Case-control	⊕⊕⊕○ Moderate	Undetected
Peng, P.[14]	1.08 (0.88-1.33)	289	Case-control	⊕⊕⊕○ Moderate	Undetected
Wu, G.[35]	1.12 (0.90-1.40)	288	Case-control	⊕⊕⊕○ Moderate	Undetected
